# Drug compatibility with various closed intravenous infusion containers

**DOI:** 10.3389/fphar.2023.1265945

**Published:** 2024-01-08

**Authors:** Chang-yu Zhu, Hui-ying Zuo, Hong-lin Li, Rong-sheng Tong

**Affiliations:** ^1^ Department of Pharmacy, Sichuan Academy of Medical Sciences, Sichuan Provincial People’s Hospital, Chengdu, Sichuan Province, China; ^2^ Personalized Drug Therapy Key Laboratory of Sichuan Province, School of Medicine, University of Electronic Science and Technology of China, Chengdu, Sichuan Province, China

**Keywords:** intravenous (i.v.) infusion containers, drug compatibility, insoluble particles, nonpolyvinyl chloride (non-PVC) infusion bags, upright polypropylene infusion bags, inner sealed polypropylene infusion bags

## Abstract

**Objective:** The aim was to systematically compare the drug compatibility with various closed intravenous (i.v.) infusion containers, to provide a reference for selecting a relatively superior infusion container and improve the medication safety for patients in clinical practice.

**Methods:** The compatibility of four commonly used clinical injections (ceftazidime, pantoprazole sodium, ambroxol hydrochloride, edaravone) with three representative closed i. v. infusion containers (non-PVC infusion bags, upright polypropylene infusion bags, inner sealed polypropylene infusion bags) prefilled with infusion fluids (0.9% sodium chloride or 5% dextrose) in the Chinese market were investigated in this study. The particle counts of both infusion fluids and diluted chemical injections by infusion fluids in various infusion containers were determined by the light obscuration method. At 0, 2 and 6 h after four injections following dilution with infusion fluids in each container, the pH of the solutions was detected, and the physical properties were examined by visual inspection. Meanwhile, the drug concentrations were assessed by high performance liquid chromatography (HPLC).

**Results:** As for either infusion fluids or diluted injections by infusion fluids, the particle counts in non-PVC infusion bags were significantly greater than those in the other two bags under some circumstances. The particle counts in diluted injections by infusion fluids increased dramatically compared with those in infusion fluids in all infusion containers, especially for the small-size particles. But pH, physical properties and drug concentrations of diluted infusion solutions in all infusion containers remained nearly unchanged over the test period.

**Conclusion:** Closed i. v. infusion containers included in this study are all well-compatible with four injections. Moreover, the closed infusion containers produced by Chinese manufacturers have met the international quality standard. Particularly, the intravenous admixture preparation process needs to be optimized to reduce the overall particulate contaminants.

## 1 Introduction

Intravenous (i.v.) infusion therapy is one of the commonly used drug delivery routes in clinical treatment. As the pharmaceutical industry develops to meet the clinical demand, the i. v. infusion system has switched from open i. v. infusion system to closed i. v. infusion system (Maki et al., 2011). Closed i. v. infusion system means that the infusion drug containers are fully self-folding that do not require or use any external vent to empty the infusion solution, which can avoid contamination of the drug solution by exogenous air microorganisms, improving the medication safety and cost-effectiveness (Franzetti et al., 2009; Tarricone et al., 2010). Closed i. v. infusion system has been widely used globally (Rosenthal and Maki, 2004), while there is still a gap in the penetration rate in China. However, as COVID-19 prevention and control on an ongoing basis was conducted in China, the closed i. v. infusion system has also gained further attention and recognition (Fakih et al., 2022).

The closed i. v. infusion system consists of closed i. v. infusion containers and administration set. In most developed countries, such as European countries and the United States, closed i. v. infusion containers are dominated by soft infusion bags (PVC infusion bags and non-PVC infusion bags), and the mainstream infusion companies (Baxter, Fresenius Kabi, B Braun) in these countries are responsible for producing soft infusion bags prefilled with infusion fluids (Pourroy et al., 2005). PVC infusion bags are infusion containers made from polyvinyl chloride film. However, non-PVC infusion bags are made from non-PVC medical packaging film, primarily three-layer or five-layer co-extrusion infusion films, such as polypropylene, polyethylene, ethylene-propylene polymerization, copolyester ether, and ethylene vinyl acetate (EVA). In China, in addition to soft infusion bags, upright polypropylene infusion bags are also widely used. Moreover, Chinese enterprises have developed inner sealed polypropylene infusion bags that can be self-folding for closed i. v. infusion in recent years. Currently, the above three types of infusion bags constitute the most commonly used closed i. v. infusion containers in China (Cui et al., 2020).

As a container holds and directly contacts drug solution, the difference in drug compatibility with infusion containers may affect the safety and efficacy of clinical medication. The drug compatibility with different infusion containers may vary due to their main materials, additive formulations, and production processes (den Brok et al., 2005; Thiesen and Krämer, 1999). Especially for the infusion fluids (0.9% sodium chloride or 5% dextrose), the quality control standard should not only conform to the relevant requirements of the Pharmacopoeia of the People’s Republic of China, but also it should ensure good compatibility with the additive drugs in various containers.

At present, some studies have reported drug compatibility with closed i. v. infusion containers, but the containers investigated in these unidimensional studies are not comprehensive, so no systematic studies are currently available (Ezquer-Garin et al., 2019; Feng et al., 2020). Most importantly, there are not any comparative studies of critical indicators of mainstream infusion containers between Chinese companies and global joint ventures. Therefore, we attempted to compare three representative closed i. v. infusion containers produced by Chinese mainstream infusion manufacturers or global joint infusion ventures in several physical and chemical indicators, to assess the compatibility of 4 commonly used clinical injections with different containers prefilled with infusion fluids. This study may provide a reference for improving patient medication safety and promoting global pharmaceutical industry development in clinical practice.

## 2 Materials and methods

### 2.1 Instruments

GWF-8JD Particle Analyzer (Tianjin Tianhe Analytical Instruments Co., Ltd.), UltiMate 3,000 High Performance Liquid Chromatography (Thermo Fisher Scientific), ULUP-III-20T Ultra Low Organic Ultrapure Water Analyzer (Sichuan ULUPure Technology Co., Ltd.), Poroshell 120 EC-C18 Chromatographic Column (Agilent Company in the United States), Excel 5 C18-Amide Chromatographic Column (ACE Company in the United Kingdom), MG II C18 Chromatographic Column (Shiseido Company in Japan) CPA225D electronic Analytical balance (Sartorius, Germany), pHS-3C pH meter (Shanghai INESA physics optical instrument Co., Ltd.), etc.

### 2.2 Medicines and reagents

Non-PVC infusion bags containing 0.9% sodium chloride (batch numbers S2105072) or 5% dextrose (batch numbers S2104038) were purchased from Shanghai Baxter Medical Products Co., Ltd, a global joint venture in China; Upright polypropylene infusion bags containing 0.9% sodium chloride (batch number C20121411) or 5% dextrose (batch number C21030208) were purchased from Sichuan Kelun Pharmaceutical Co., Ltd, one domestic manufacturer in China; Inner sealed polypropylene infusion bags containing 0.9% sodium chloride (batch number 2007300404) or 5% dextrose (batch number 210117402) were purchased from Wuhan Binhu Double-Crane Pharmaceutical Co., Ltd, another domestic manufacturer in China.

Ceftazidime injections (batch number 2010211, 1.0g) were purchased from Hainan Hailing Chemical Pharmaceutical Co., Ltd); Pantoprazole sodium injections (batch number 20061911, 40 mg) were purchased from Ruiyang Pharmaceutical Co., Ltd; Ambroxol hydrochloride injections (batch number 925364, 2 mL: 15 mg) were purchased from Boehringer-Ingelheim); Edaravone injections (batch number 2011094, 20 mL: 30 mg) were purchased from Guorui Pharmaceutical Co., Ltd).

Ceftazidime (batch number: 130,484-201806, content: 85.80%), pantoprazole sodium (batch number: 100,575-201505, content: 95.80%), ambroxol hydrochloride (batch number: 100,599-201905, content: 99.90%) and edaravone (batch number: 100,620-201703, content: 100.00%) standard products were purchased from the China Institute for Food and Drug Control; Both methanol and acetonitrile were of chromatographic grade.

### 2.3 Methods

The following methods used in this study referred to the Chinese Pharmacopoeia 2020 edition, which was reviewed and approved by the National Medical Products Administration (NMPA) and the National Health Commission of the People’s Republic of China in July 2020. This updated edition reflects not only the current level of technology used in the pharmaceutical industry in China but also the technologies used for international drug quality control (Xu et al., 2021).

#### 2.3.1 Quantification of insoluble particles

Pharmacopoeias of various countries have imposed strict requirements on particle counts in injections. Two types of methods in insoluble particulate matter test are light obscuration method and microscopic particle count method, and the limits of insoluble particles for different detection methods are shown in Table 1 (Harazono et al., 2019). In 2010, the Chinese Pharmacopoeia ChP2010 was updated, and the requirements for the limits of insoluble particles for injections under different detection methods have been harmonized with the international standards. Currently, the pharmacopoeias of various countries only control insoluble particles with a particle size of 10 μm or more in injections, but do not have clear requirements for particles with a particle size of less than 10 μm.

**TABLE 1:** Comparison of restriction on insoluble particulate matter test for injections (volume≥100 mL) in pharmacopoeias of different countries.

Pharmacopoeias	Light obscuration		Microscopic particle count	
	≥10 μm	≥25 μm	≥10 μm	≥25 μm
Chinese Pharmacopoeia, ChP2020	≤25 number·mL-1	≤3 number·mL-1	≤12 number·mL-1	≤2 number·mL-1
United States Pharmacopoeia, USP 43-NF 38	≤25 number·mL-1	≤3 number·mL-1	≤12 number·mL-1	≤2 number·mL-1
European Pharmacopoeia, EP 10.0	≤25 number·mL-1	≤3 number·mL-1	≤12 number·mL-1	≤2 number·mL-1
British Pharmacopoeia, BP 2021	≤25 number·mL-1	≤3 number·mL-1	≤12 number·mL-1	≤2 number·mL-1
Japanese Pharmacopoeia, JP 18	≤25 number·mL-1	≤3 number·mL-1	≤12 number·mL-1	≤2 number·mL-1

The quantification of insoluble particulate matter was performed by the light obscuration method strictly according to “Part IV 0903 Insoluble Particle Inspection Method” of the 2020 edition of the Chinese Pharmacopoeia. In accordance with the clinical dosage in the package inserts, Ceftazidime and Pantoprazole sodium were reconstituted and further diluted in three infusion bags containing 0.9% sodium chloride, Edaravone was diluted in three infusion bags containing 0.9% sodium chloride, and Ambroxol hydrochloride was diluted in three infusion bags containing 5% dextrose, respectively. All the infusion solutions were placed for 20 min until there were no air bubbles. Then the container was carefully opened and placed on the sampler of the instrument to extract 5 mL of solution, and the counts of particles in the micrometers size range (2–5 um, 5–10 um, 10–25 um and ≥25 um) were measured. A total of five replicate samples were tested, and the average value of four results was calculated after discarding the first result. All the operations were conducted by one nursing staff in a class II cleanroom.

#### 2.3.2 Concentration determination

At 0, 4 and 6 h after four injections following dilution by infusion fluids in different containers, the concentration of ambroxol hydrochloride was determined by an HPLC assay according to the standard of National Medical Products Administration of China (YBH03762018) and the general rule for HPLC in the Pharmacopoeia of the People’s Republic of China (Part IV, 0512 HPLC): a chromatographic column packed with octadecylsilane chemically bonded to silica gel was used, a mixture of 0.01 mol/L ammonium hydrogen phosphate solution, acetonitrile and methanol (50:40:10) was selected as mobile phase, and the detective wavelength was 248 nm.

The concentration of ceftazidime, pantoprazole and edaravone was measured by HPLC assay based on the corresponding chapter of Pharmacopoeia of the People’s Republic of China (Part II) and the general rule for HPLC in the Pharmacopoeia of the People’s Republic of China (Part IV, 0512 HPLC): a chromatographic column packed with octadecylsilane chemically bonded to silica gel was used; the mobile phase for ceftazidime injection consisted of a mixture of acetonitrile, pH 7.0 phosphate buffer and water (40:200:1760), and the detective wavelength was 254 nm; the mobile phase for pantoprazole sodium injection consisted of a mixture of 0.01 mol/L potassium dihydrogen phosphate solution and acetonitrile (65:35), and the detective wavelength was 289 nm; the mobile phase for pantoprazole sodium injection consisted of a mixture of methanol and 0.05 mol/L ammonium dihydrogen phosphate solution (50:50), and the detective wavelength was 245 nm.

The initial drug concentrations were defined as 100%, and the concentrations at 4 and 6 h were expressed as the percentage of the initial concentration. Six replicate injections of solutions were used in each test.

#### 2.3.3 pH measurement

The pH values were monitored according to the 2020 edition of the Pharmacopoeia of the People’s Republic of China (Part IV 0631 pH Measurement Method): prior to the measurement by a pH meter, two standard pH calibration solutions were selected to calibrate the instrument to ensure that the pH value of the sample was between them. Then each test sample in duplicate was prepared at predefined time intervals (0, 2 and 6 h) and mean pH values were calculated.

#### 2.3.4 Characterization of physical properties

Physical properties such as color and turbidity for diluted ambroxol hydrochloride infusion solutions were measured by visual inspection according to the standard YBH03762018 of China’s National Medical Products Administration, and property measurements for diluted ceftazidime, pantoprazole sodium and edaravone infusion solutions were performed in accordance with Part II of the Pharmacopoeia of the People’s Republic of China (2020). The samples were observed by visual inspection against a white background under a good light and the results were recorded.

### 2.4 Statistical analysis

Minitab statistical software was used to perform data analysis. Anderson-Darling test indicated that the data followed normality distribution, which were expressed as mean ± standard deviation. The homogeneity of variance was checked by Bartlett test. If the variance was equal, the data were analyzed with one-way ANOVA followed by Tukey test. If the variance was unequal, Welch test together with Games-Howell *post hoc* test was used for data analysis.

## 3 Results

### 3.1 Particle counts of infusion fluids (0.9% sodium chloride or 5% dextrose) in various infusion containers

As shown in Table 2, the number of insoluble particles with particle sizes of 2–5, 5–10 and 10–25 μm in 0.9% sodium chloride stored in non-polyvinyl chloride (non-PVC) infusion bags was significantly higher than that in upright polypropylene infusion bags and inner sealed polypropylene infusion bags (*p* < 0.05). However, there was no statistical difference in particle counts among three closed i. v. infusion bags containing 5% dextrose.

**TABLE 2:** Particle counts of infusion fluids (0.9% sodium chloride or 5% dextrose) in various infusion containers.

Vehicles	Containers	Particle size range/(number·mL^-1^)			
		2–5 μm	5–10 μm	10–25 μm	≥25 μm
0.9% sodium chloride	non-polyvinyl chloride	22.10 ± 3.60	8.860 ± 1.210	0.980 ± 0.356	0.0
	upright polypropylene	3.16 ± 1.88^*^	0.340 ± 0.313^*^	0.080 ± 0.084^*^	0.0
	inner sealed polypropylene	1.96 ± 1.86^*^	0.400 ± 0.524^*^	0.160 ± 0.207^*^	0.0
5% dextrose	non-polyvinyl chloride	13.28 ± 6.20	3.76 ± 2.71	0.80 ± 0.758	0.0
	upright polypropylene	14.48 ± 3.97	2.660 ± 0.594	0.400 ± 0.235	0.0
	inner sealed polypropylene	17.94 ± 3.67	2.740 ± 1.014	0.220 ± 0.217	0.0

Note: * represents *p* < 0.05 for comparison with the non-polyvinyl chloride bags.

### 3.2 Particle counts of diluted infusion solutions in various infusion containers

After four chemical injections were diluted by infusion fluids in various containers, there was a trend toward higher particle counts with particle size of 2–5 μm in diluted ceftazidime injection stored in non-PVC infusion bags containing 0.9% sodium chloride, compared with the upright polypropylene infusion bags and inner sealed polypropylene infusion bags (*p* < 0.05), so are the particles with particle size of 5–10 μm in diluted ambroxol hydrochloride injection by 5% dextrose (*p* < 0.05), and no statistical difference in particle counts was observed in other groups (Table 3).

**TABLE 3:** The particle counts of diluted 4 different injections in various infusion containers prefilled with infusion fluids.

Injections	Vehicles	Containers	Particle size range/(number·mL^-1^)			
			2–5 μm	5–10 μm	10–25 μm	≥25 μm
Ceftazidime	0.9% sodium chloride	non-polyvinyl chloride	651.7 ± 105.4	152.3 ± 27.9	2.90 ± 0.616	0.0
		upright polypropylene	424.8 ± 182.8^*^	113.3 ± 46.1	3.00 ± 2.006	0.2
		inner sealed polypropylene	420.3 ± 99.3^*^	96.9 ± 36.5	2.12 ± 0.996	0.2
Pantoprazole	0.9% sodium chloride	non-polyvinyl chloride	326.0 ± 76.3	102.9 ± 40.5	2.82 ± 0.259	0.0
		upright polypropylene	303.4 ± 94.7	77.6 ± 30.9	1.98 ± 0.691	0.0
		inner sealed polypropylene	379.7 ± 53.2	112.9 ± 42.4	2.26 ± 1.133	0.1
Edaravone	0.9% sodium chloride	non-polyvinyl chloride	79.3 ± 33.9	21.44 ± 12.56	1.32 ± 0.876	0.0
		upright polypropylene	89.08 ± 13.55	26.80 ± 5.37	1.88 ± 0.259	0.1
		inner sealed polypropylene	98.4 ± 35.2	36.28 ± 8.88	2.16 ± 0.513	0.0
Ambroxol	5% dextrose	non-polyvinyl chloride	157.7 ± 57.8	42.9 ± 23.5	0.78 ± 0.602	0.0
		upright polypropylene	74.44 ± 19.92	19.68 ± 8.96*	0.82 ± 0.396	0.0
		inner sealed polypropylene	84.7 ± 16.75	17.10 ± 7.89*	0.562 ± 0.437	0.0

Note: * represents *p* < 0.05 for comparison with the non-polyvinyl chloride bags.

### 3.3 Stability of chemical injections after dilution by infusion fluids in various infusion containers

**FIGURE 1:**
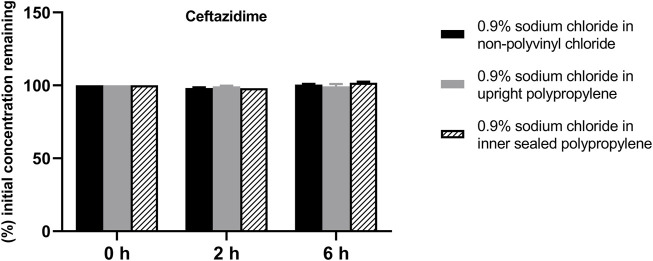
Drug concentrations of diluted ceftazidime injection in various infusion containers prefilled with infusion fluids. The initial drug concentrations were defined as 100%, and the concentrations at 4 and 6 h were expressed as the percentage of the initial concentration. Six replicate injections of solutions were used in each test.

**FIGURE 2:**
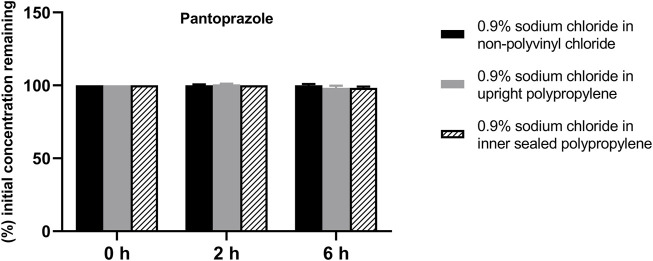
Drug concentrations of diluted pantoprazole sodium injection in various infusion containers prefilled with infusion fluids. The initial drug concentrations were defined as 100%, and the concentrations at 4 and 6 h were expressed as the percentage of the initial concentration. Six replicate injections of solutions were used in each test.

**FIGURE 3:**
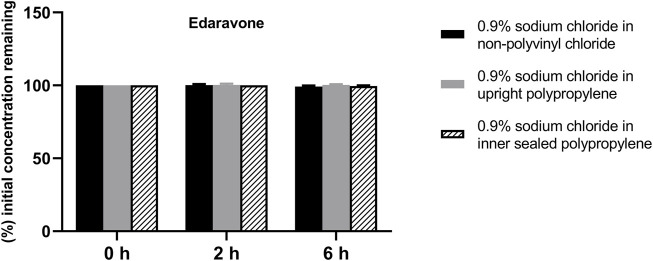
Drug concentrations of diluted edaravone injection in various infusion containers prefilled with infusion fluids. The initial drug concentrations were defined as 100%, and the concentrations at 4 and 6 h were expressed as the percentage of the initial concentration. Six replicate injections of solutions were used in each test.

**FIGURE 4:**
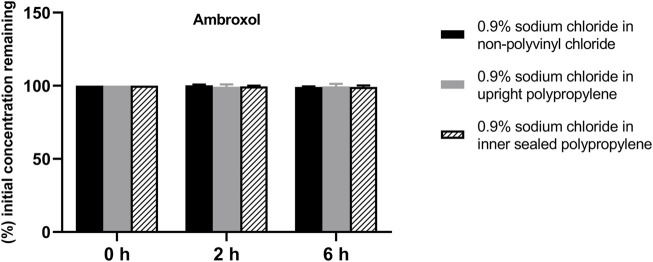
Drug concentrations of diluted ambroxol hydrochloride injection in various infusion containers prefilled with infusion fluids. The initial drug concentrations were defined as 100%, and the concentrations at 4 and 6 h were expressed as the percentage of the initial concentration. Six replicate injections of solutions were used in each test.

The solution pH, physical properties and drug concentrations in the diluted infusion solution were measured after 0, 2 and 6 h of storage. The pH fluctuation was less than 0.1 over the 6-h observation period, and no turbidity or colour changes were observed during the observation period of 6 h (Table 4). Drug concentrations declined less than 2% over the entire test period (Figures 1–4). So the chemical injections after dilution by infusion fluids were physicochemically stable for a minimum of 6 h.

**TABLE 4:** pH and physical property of diluted 4 different injections in various infusion containers prefilled with infusion fluids.

Injections	Vehicles	Containers	pH			Physical property (0 h/2 h/6 h)
			0 h	2 h	6 h	
Ceftazidime	0.9% sodium chloride	non-polyvinyl chloride	6.77	6.77	6.87	Pale yellow transparent
		upright polypropylene	6.90	6.85	6.97	Pale yellow transparent
		inner sealed polypropylene	6.69	6.71	6.72	Pale yellow transparent
Pantoprazole	0.9% sodium chloride	non-polyvinyl chloride	9.28	9.28	9.28	Colorless and transparent
		upright polypropylene	9.23	9.23	9.23	Colorless and transparent
		inner sealed polypropylene	9.25	9.24	9.25	Colorless and transparent
Edaravone	0.9% sodium chloride	non-polyvinyl chloride	4.15	4.14	4.12	Colorless and transparent
		upright polypropylene	4.17	4.19	4.17	Colorless and transparent
		inner sealed polypropylene	4.18	4.17	4.14	Colorless and transparent
Ambroxol	5% dextrose	non-polyvinyl chloride	4.74	4.73	4.69	Colorless and transparent
		upright polypropylene	4.95	4.95	4.96	Colorless and transparent
		inner sealed polypropylene	5.32	5.28	5.28	Colorless and transparent

## 4 Discussion

In our study, all preparation of intravenous admixture was operated in a class II biosafety cabinet to strictly control the cleanliness of the operation environment and reduce the interference of environmentally insoluble particles with the results (American Society of Health System Pharmacists, 2014). And the intravenous admixture preparation process was performed by the same nursing staff to reduce the test error caused by personal operation and to ensure objective and accurate data in each test. Moreover, standardized nursing operations were adopted to prepare the reconstituted medication, such as choosing small side port needles for vertical puncture; limiting the puncture times and sites of injection stopper/gasket (Higgins, 2005); cutting the glass ampoule with a grinding wheel for 1/4 circle, disinfecting it with a medical alcohol cotton ball, and snapping open the ampoule at the neck by hand (Lavery, 2011). The above approaches aim to avoid errors caused by the environment and operation methods as much as possible. In addition, the inspection periods were set to cover the time for nurses to prepare intravenous admixture, temporary storage time and infusion time. Therefore, the observation time points for pH, concentrations and physical properties were set at 0, 2 and 6 h after admixture preparation in this study, which has clinical significance (Sugiyama et al., 2016).

Insoluble particles are non-metabolizable particulate impurities that are insoluble in the infusion solution, invisible to the naked eye, and usually less than 50 μm in size. Insoluble particles can cause a series of hazards such as phlebitis, granuloma, and vascular embolism when they are delivered into the human body through the venous vessels (Larson et al., 1984). Some researchers have pointed out that the excessive insoluble particles in infusion solutions are the most important factor triggering the occurrence of adverse reactions during infusion (Perez et al., 2016). Through comparing Table 2 with Table 1, we found that the particle control of all three closed i. v. infusion containers prefilled with infusion fluids met and even exceeded the requirements of the 2020 edition of the Chinese Pharmacopoeia, and the particle control of two closed i. v. infusion containers prefilled with infusion fluids from Chinese manufacturers met and even exceeded the requirements of European, American and Japanese pharmacopoeias. Especially, the infusion containers of Chinese manufacturers were superior to those of global joint ventures in terms of insoluble particle counts in some size ranges. However, this study did not demonstrate that inner sealed polypropylene infusion bags contained significantly less insoluble particles than upright polypropylene infusion bags, which was inconsistent with a prior research (Li et al., 2019). It was inferred that the total particle counts were mainly influenced by the manufacturing process control of each manufacturer, rather than the form of infusion containers (Duchek and Havasi, 2018). After admixture preparation in Table 3, the number of insoluble particles all increased significantly, especially the number of small-size particles (2–10 μm) increased tens or even hundreds of times compared with that before admixture preparation (Table 2), consistent with other reports (Feng et al., 2020). It was suggested that the inter-product differences of infusion containers prefilled with infusion fluids contribute very little to the particle counts in intravenous admixture, but they are attributed to other various factors, such as drug properties investigated in this study, as well as the clinical operation methods, operational environment and storage time after admixture preparation reported in other studies (American Society of Health System Pharmacists, 2014; Perez et al., 2015; Ayres, 2018). Consequently, it is more important to strengthen the management of the whole process in clinical practices to control the particle counts in intravenous admixture, such as standardized operation, centralized admixture preparation in pharmacy intravenous admixture services (PIVAS), information management and the application of precise filtering infusion apparatus if necessary (Négrier et al., 2021).

The drug compatibility of i. v. infusion containers refers to migration or adsorption may occur between the drug and the container, which affects the quality and safety of the drugs (Palmgrén et al., 2006). Previous studies have reported that PVC infusion bags have certain adsorption properties for a variety of drugs, so the choice of this kind of container should be avoided for clinical practices (Peters and Hayball, 1990). As the pharmaceutical industry develops, PVC infusion bags are replaced by non-PVC infusion bags without adding bis(2-ethylhexyl) phthalate (DEHP), and new closed i. v. infusion containers have been developed gradually. This study shows that three representative closed i. v. infusion containers currently available in the Chinese market did not alter the pH, physical properties and drug concentrations of commonly used chemical drugs in intravenous admixture (Table 4; Figures 1–4), which obtained similar stability results with other oversea reports. For instance, Monique W. J. den Broka showed that when Imexon was diluted in soft infusion bag (Baxter) prefilled with 0.9% sodium chloride and, the pH value changed from 6.0 to 7.1 and concentration of Imexon decreases 1.7% after storage for 2 h, thus the infusion solutions were stable for 2 h in Baxter infusion bag (den Brok et al., 2005). André Mohr studied the stability of thiamazole (methimazole) diluted in prefilled 0.9% sodium chloride soft infusion bags (Fresenius Kabi), and showed that the pH values remained nearly unchanged, no evidence of colour change was observed, and the concentration decreased approximately 1% within 24 h (Mohr and Krämer, 2022). Different drugs have varying properties, but the results of these studies were similar, which indicate that the infusion containers produced by Chinese companies in this study have the same good drug compatibility with oversea products. However, our study has some limitations, because the compatibility with other types of drugs such as biosimilars and new drug carriers needs more exploration.

In conclusion, all three representative closed i. v. infusion containers from Chinese mainstream infusion manufacturers and global joint infusion ventures included in this study are well compatible with commonly used clinical chemical injections and can be applied in clinical practices. Moreover, the whole process control of intravenous admixture preparation and infusion should be strengthened to minimize the risk of a significant increase of insoluble particles in intravenous admixture due to various factors. Furthermore, this study reported for the first time that the quality control of infusion containers prefilled with infusion fluids produced by Chinese mainstream infusion manufacturers has met the international quality standard.

## Data Availability

The original contributions presented in the study are included in the article/Supplementary material, further inquiries can be directed to the corresponding author.
